# Allelopathic Effects of Compounds from the Ethanol Extract of *Artemisia frigida* on Five Invasive Alien Plants

**DOI:** 10.3390/plants15101528

**Published:** 2026-05-16

**Authors:** Nufen Li, Jiadi Zhang, Wei Hua, Lifeng Wang, Shangfeng Zhou, Kailin Liu, Haona Yang

**Affiliations:** 1 College of Plant Protection, Hunan Agricultural University, Changsha 410128, China; nufenli1743@163.com (N.L.); 18439726735@163.com (J.Z.); weiweihua0523@163.com (W.H.); 2 Hunan Institute of Plant Protection, Hunan Academy of Agricultural Sciences, Changsha 410125, China; ifwang@hunaas.cn (L.W.); sfzhou7389@hunaas.cn (S.Z.); 3 Yuelushan Laboratory, Changsha 410128, China

**Keywords:** invasive alien plants, *Artemisia frigida*, ethanol extract, Allelochemicals, 4-Ethyloctanoic acid

## Abstract

Invasive alien plants seriously threaten native plant biodiversity and agricultural production. The development of environmentally friendly agriculture requires sustainable weed control techniques to manage these invasive alien weeds. This study evaluated the allelopathic effects of ethanol extract from *Artemisia frigida* against five invasive alien plants (*Ageratum conyzoides*, *Bidens pilosa*, *Ipomoea purpurea*, *Eclipta prostrata*, and *Amaranthus retroflexus*). The main components in the extract were identified using high-performance liquid chromatography–tandem mass spectrometry (LC-MS/MS), and we assessed their allelopathic effects on seed germination of the five species. The results showed that the ethanol extract of *A. frigida* completely inhibited seed germination of all five invasive plants at 5 g·L^−1^. Thirteen components were identified, among which 4-ethyloctanoic acid, cis-jasmone, and p-anisic acid exhibited significant inhibitory effects. Notably, 4-ethyloctanoic acid demonstrated broad-spectrum herbicidal activity. At 50 mg·L^−1^, it completely inhibited *B. pilosa* growth and had the strongest inhibitory effects on *A. conyzoides* and *E. prostrata*. This compound disrupted redox homeostasis and induced oxidative stress by modulating antioxidant enzyme activities, including superoxide dismutase (SOD), peroxidase (POD), and catalase (CAT). These findings indicate that 4-ethyloctanoic acid is the main allelochemical with herbicidal potential in *A. frigida*, providing a theoretical basis for developing novel herbicides and environmentally friendly control techniques for invasive alien plants.

## 1. Introduction

Biological invasions are one of the top five direct drivers of global biodiversity decline [1], have caused enormous ecological, economic and evolutionary impacts worldwide, and pose a serious threat to biodiversity conservation [2]. Among these invasions, invasive alien plants have become a major global ecological security issue and a significant component of global environmental change [3]. Invasive alien plants rapidly colonize new habitats by virtue of their higher growth rates, competitive success, and allelopathic effects, resulting in reduced crop yields and increased management costs [4,5]. For example, *Ageratum conyzoides*, *Bidens pilosa*, *Ipomoea purpurea*, *Eclipta prostrata*, and *Amaranthus retroflexus* are notorious global invasive alien plants, widely distributed in crop fields and orchards across tropical, subtropical, and temperate regions [6,7,8,9]. *A. conyzoides* secretes various allelochemicals, such as protocatechuic acid, gallic acid, p-coumaric acid, p-hydroxybenzoic acid, and ferulic acid, which inhibit the germination and seedling growth of competing plant species and thus enhance its invasiveness in agricultural ecosystems [8]. The seeds of *B. pilosa* possess 2–3 barbed spines, which can attach to human clothing and animal fur, enabling long-distance dispersal through the movement of humans and animals, thereby further accelerating their spread and reproduction. *B. pilosa* impacts agricultural productivity by infesting 31 economically vital crops across over 40 countries [10]. *E. prostrata* is a problematic weed in several crops, including peanut, soybean, rice, sugarcane, and corn. In China, rice yield losses due to *E. prostrata* infestation have been reported to range from 4% to 70% [11]. *I. purpurea* generates a dense canopy by climbing on mature trees, shrubs, and other plant species, competing with crops and native plants for nutrients, water, and solar radiation [12]. Similarly, *A. retroflexus*, as a C4 plant, is constantly competing with crops for nutrients, water, and light, leading to severe yield losses in soybean (up to 58%), corn (5–34%), and other crops [9].

Herbicide is the primary strategy for weed control owing to its ease of application, high efficacy, and cost-effectiveness. However, prolonged and repeated use of herbicides has led to the continuous evolution and spread of herbicide-resistant weed populations worldwide. According to the International Herbicide-Resistant Weed Survey, there are currently 541 unique cases (species x site of action) of herbicide-resistant weeds globally, with 273 species including 156 dicots and 117 monocots (www.weedscience.org). Among these, *A. conyzoides* has been confirmed as resistant to acetolactate synthase (ALS) inhibitor herbicides. The population of *A. conyzoides* in the sugarcane field exhibited an average resistance ratio (R/S) of 54-fold to metsulfuron compared with the roadside area in Australia [13]. A target-site mutation results in broad-spectrum cross-resistance to ALS inhibitors in resistant *E. prostrata* populations, causing field rates of ALS inhibitors to fail in controlling this weed in some farmlands in China [14]. Furthermore, a mutation conferring resistance to PSII-inhibiting herbicides has been reported in *A. retroflexus* [15]. The banning of certain high-risk herbicides has further complicated weed control. Therefore, it is imperative to develop sustainable and environmentally friendly weed management strategies.

Allelopathy refers to any direct or indirect effect (harmful or beneficial) by a plant or microorganism on another plant through the production of chemical compounds released into the environment [16]. This phenomenon provides a new perspective for the development of sustainable weed control strategies [17]. Natural product-derived herbicides offer unique advantages, including high efficacy, low toxicity, enhanced crop safety, and good environmental compatibility. Moreover, they pose a low risk of resistance development [18]. Consequently, they represent a promising new direction for the development of novel herbicides. *A. frigida*, a perennial herbaceous plant of the *Asteraceae* family, is known to produce abundant volatile organic compounds, with 1,8-cineole and camphor as major constituents, as well as sesquiterpene lactones such as achillin in its aqueous extracts [19]. The allelopathy of *A. frigida* has been recognized as a key mechanism contributing to its dominance in overgrazed and degraded grasslands. Studies have shown that aqueous extracts of *A. frigida* inhibited seed germination and seedling growth of *Leymus chinensis*, *Stipa krylovii*, and *Cleistogenes squarrosa* [20,21]. This study aims to screen the main allelochemicals from the ethanol extract of *A. frigida* and to evaluate their inhibitory effects on these five invasive alien plants (*A. conyzoides*, *B. pilosa*, *I. purpurea*, *E. prostrata*, and *A. retroflexusas*). These allelochemicals could be used for developing plant-derived herbicides, thereby providing both theoretical foundations and technical support for the sustainable management of invasive alien plants.

## 2. Results

### 2.1. Effects of Ethanol Extract of A. frigida on Seed Germination of Five Invasive Alien Plants

**Figure 1:**
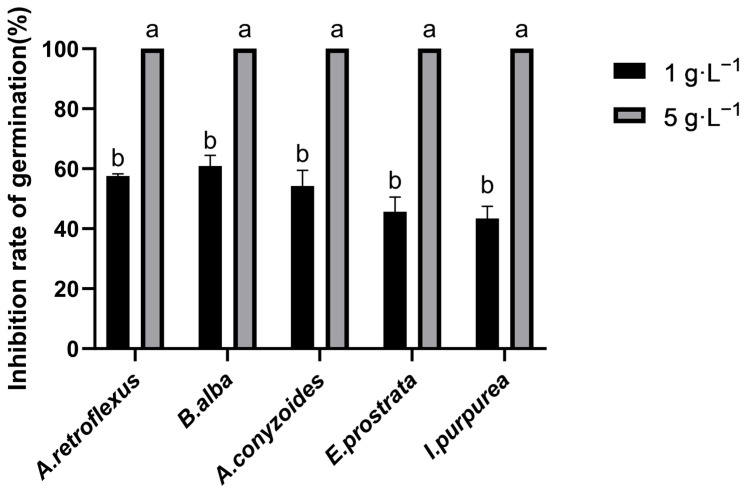
Effects of ethanol extract of *A. frigida* on seed germination of five invasive alien plants. Different letters indicate statistically significant differences between experimental groups (*p* < 0.05).

The ethanol extract of *A. frigida* inhibited the germination of five invasive alien plants in a concentration-dependent manner, with the inhibitory effect increasing significantly as the extract concentration increased (Figure 1). At 1 g·L^−1^, the inhibition rates of the tested five species ranged from 44% to 62%, among which *B. alba* exhibited the highest inhibition rate (61.57%), while *I. purpurea* showed a relatively lower inhibition rate (44.47%). When the concentration was increased to 5 g·L^−1^, the germination inhibition rates of all tested species reached 100%, indicating complete inhibition of seed germination. To identify the active compounds for this allelopathic inhibition, the chemical constituents of the *A. frigida* ethanol extract were subsequently analyzed and characterized.

### 2.2. Analysis and Identification of Compounds in A. frigida Ethanol Extract

The chemical composition of the ethanol extract of *A. frigida* was analyzed using LC-MS/MS, identifying over 1700 compounds, including fatty acids, terpenoids, alkaloids, polyketides, shikimic acid, and phenylpropanoids. Based on MS2 score-based qualitative matching, 1 major compound was identified (Table 1). Including four fatty acids: 4-ethyloctanoic acid, dodecanoic acid, adipic acid, and cis-jasmone. Six shikimic acid derivatives and phenylpropanoids: 3,5-dimethoxycinnamic acid, 4′-hydroxyacetophenone, 3-hydroxyacetophenone, 3,4-dihydroxybenzoic acid, p-anisic acid, and 4-hydroxybenzaldehyde. One alkaloid compound, 3-(2,5-dimethoxyphenyl) propionic acid, one terpenoid compound, (+)-camphor, one amino acid and short peptide compound, N-acetylvaline, were identified.

### 2.3. Effects of Compounds of A. frigida on Seed Germination of Five Invasive Alien Plants

Among the 16 compounds tested at 0.1 g·L^−1^, 0.5 g·L^−1^, and 1 g·L^−1^, 4-ethyloctanoic acid (L1), cis-jasmone (L4) and p-anisic acid (L12) were found to inhibit the germination of at least one or several receptor plant species at 0.1 g·L^−1^ (Table 2). Among these, L1 completely inhibited the seed germination of *B. alba*, *A. retroflexus*, and *E. prostrata*. L4 also completely inhibited the seed germination of *B. alba*, *A. retroflexus*, and *E. prostrata*. L12 completely inhibited the seed germination of *B. alba*. The remaining compounds showed no significant effects on the germination rates of the seeds of the five alien invasive plants.

### 2.4. Effects of Four Allelochemicals on Seedling Growth of Five Invasive Alien Plants

**Figure 2:**
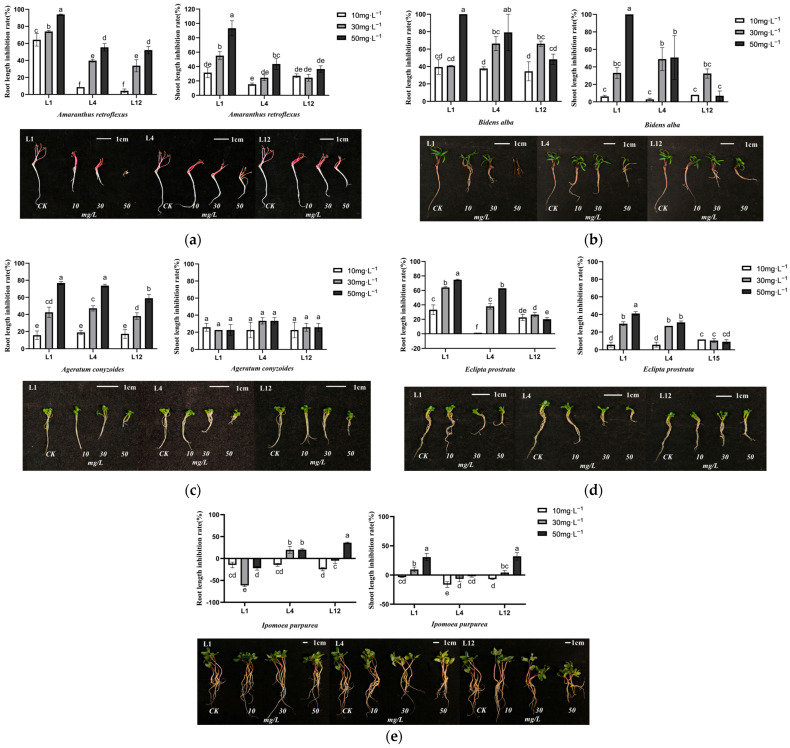
Effects of four allelochemicals on seedling growth of five invasive alien plants. (**a**) *A. retroflexus*; (**b**) *B. alba;* (**c**) *A. conyzoides;* (**d**) *E. prostrata;* (**e**) *I. purpurea*. Different letters indicate significant differences between different treatments (*p* < 0.05).

L1, L4 and L12 exhibited different inhibitory effects on the seedling growth of these five alien invasive plants. L1 significantly reduced the root length and shoot length of *A. retroflexus*, *B. alba*, and *E. prostrata* (Figure 2a,b,d), with complete inhibition of *B. alba* growth observed at a concentration of 50 mg·L^−1^. L4 showed the strongest inhibitory effect on *A. conyzoides* (Figure 2c), and L12 showed the strongest inhibitory effect on *I. purpurea*, but the effect is not statistically significant (Figure 2e). In conclusion, L1 exhibited broad-spectrum inhibitory activity against multiple species and was identified as the main allelochemical for further investigation.

### 2.5. Effect of 4-Ethyloctanoic Acid on Enzyme Activities of Five Invasive Alien Plants

**Figure 3:**
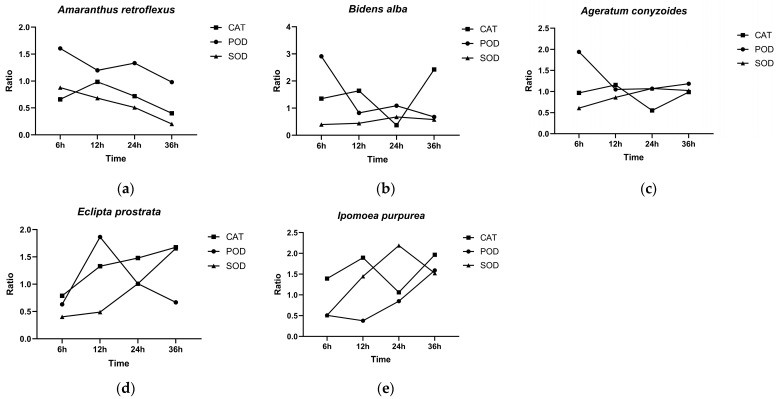
Effects of 4-ethyloctanoic acid treatment on the activities of antioxidant enzymes of five invasive alien plants. The ratio is the average enzyme activity of the treatment group divided by the average enzyme activity of the control group at the same time. (**a**) *A. retroflexus*; (**b**) *B. alba*; (**c**) *A. conyzoides*; (**d**) *E. prostrata*; (**e**) *I. purpurea*.

Treatment with 5 g·L^−1^ of 4-ethyloctanoic acid induced distinct patterns of SOD, POD, and CAT activities among the five invasive alien plants, with most antioxidant enzymes showing altered activity at various time points relative to the controls. A ratio > 1 indicates higher enzyme activity in the treatment group than in the control group, whereas a ratio < 1 indicates greater inhibition of enzyme activity in the treatment group. Based on their antioxidant responses under L1 treatment, the five invasive alien plants showed different strategies: (1) In *A. retroflexus*, the early antioxidant response was dominated by POD, while CAT and SOD activities were strongly suppressed (Figure 3a). (2) In *B. alba*, POD activity was most markedly enhanced at 6 h, reaching 2.91 times that of the control, indicating rapid early activation of the POD-mediated antioxidant defense system. By 36 h, CAT activity had replaced POD as the primary H_2_O_2_-scavenging enzyme, whereas SOD activity remained consistently suppressed (Figure 3b). (3) In *A. conyzoides*, the activity ratios of SOD and CAT remained near 1 throughout the treatment period, while POD was consistently upregulated, particularly at 6 h, suggesting its predominant role in mitigating L1-induced oxidative stress (Figure 3c). (4) In *E. prostrata*, during 6–12 h, the plant primarily relied on POD activity. From 24 to 36 h, CAT and SOD activities were continuously enhanced, collectively establishing a stable antioxidant defense network (Figure 3d). (5) In *I. purpurea*, CAT dominated the early-stage antioxidant defense; SOD peaked at 24 h as the mid-stage core enzyme; and CAT and POD synergistically maintained high activity in the late stage. At 36 h, the activities of CAT, SOD, and POD were all significantly higher than those in the control group (ratio > 1), reflecting adaptive regulation of the antioxidant system (Figure 3e).

## 3. Discussion

*A. frigida*, a typical member of the Asteraceae family, is a dominant plant in degraded grasslands under moderate to severe grazing intensity in northern China. Its allelopathic effect is considered the key mechanism driving this process. Previous studies have shown that the allelopathic effect of *A. frigida* significantly promoted its own biomass while inhibiting that of *Stipa krylovii*, and its aqueous extract of *A. frigida* significantly affected the aboveground, belowground, and total biomass of *Medicago sativa* and *Melilotus officinalis* [22]. Moreover, the allelopathic effect of *A. frigida* played a major role in inhibiting the belowground biomass of *Leymus chinensis* (23.97%) and *Cleistogenes squarrosa* (58.27%), while allelopathy and resource competition from *A. frigida* promoted the belowground biomass (45.12%) and aboveground biomass (46.63%) of *Potentilla acaulis* [21]. Based on previous research, this study further explores the effect of *A. frigida* on the germination of seeds from five invasive alien plants. It was found that the ethanol extract of *A. frigida* completely inhibited the seed germination of five invasive alien plants at the concentration of 5 g·L^−1^.

The identification of the main allelochemicals in the ethanol extract of *A. frigida* provides evidence for its strong allelopathic potential, as demonstrated by chemical composition analysis and bioactivity assays. LC-MS/MS analysis revealed multiple secondary metabolites in the ethanol extract of *A. frigida*. Among them, the compounds with the higher relative contents included p-anisic acid (L12, 2.19%), camphor (L9, 0.53%), and 4-ethyloctanoic acid (L1, 0.4%). This finding is inconsistent with previous results, as the chemical composition of the extract varied significantly depending on the extraction solvent used [23]. The present study found that camphor (L9) exhibited no significant allelopathic inhibitory effect on the five invasive weed species tested, which contrasts with the findings of Zhao et al. [24]. This inconsistency may be due to species-specific differences in the allelopathic response to *A. frigida* [22]. This study revealed that 4-ethyloctanoic acid, cis-jasmone, and p-anisic acid exhibited strong allelopathic effects against five invasive alien plants. Among them, 4-ethyloctanoic acid is a medium-chain branched fatty acid with a pronounced mutton-like odor, primarily used as a food flavoring agent and a key component in formulations to impart the characteristic flavor of lamb [25]. To date, its allelopathic activity has not been reported. cis-Jasmone, a derivative of jasmonic acid, has been shown to participate in chemical communication and defense responses between plants, playing a role in plant-induced defense mechanisms [26]. p-Anisic acid, a phenolic acid compound, is widely present in plant residues and rhizosphere soils. Previous studies have confirmed that p-anisic acid is one of the key allelopathic substances responsible for inhibiting seed germination and seedling growth of vegetables, such as lettuce, tomato, and pepper, in processing residues of Guayule [27].

Plants activate antioxidant defense systems under stress conditions to eliminate excess reactive oxygen species (ROS), with CAT, POD, and SOD being key antioxidant enzymes [28]. In this study, L1 treatment inhibited SOD activity in A. retroflexus. As the first line of defense against superoxide anion radicals (O_2_^−^), impairment of SOD activity directly leads to ROS accumulation and oxidative stress, which is consistent with the inhibitory effect of L1 on seed germination and growth. This result is consistent with the allelopathic effect reported by Talukder, in which benzoxazolinone (BOA) severely inhibited SOD activity in lettuce [29]. Both *E. prostrata* and *I. purpurea* established a stable defense system composed of CAT, SOD, and POD at 36h, and the synergistic action of these enzymes is crucial for plants to maintain high antioxidant levels, effectively scavenge excess ROS, and resist stress [30]. POD plays a predominant role in early responses to stress in various plants, including *B. alba* and *A. conyzoides*, where it primarily mediates stress defense. In addition to its involvement in H_2_O_2_ scavenging, POD is also implicated in cell wall lignification, making it a rapid response enzyme for stress resistance in plants [31]. In conclusion, 4-ethyloctanoic acid treatment disrupts the redox balance in the cells of weeds and induces differential antioxidant enzyme responses across species, which may enhance its herbicidal activity by overwhelming the weeds’ stress defense systems. However, the precise molecular targets of 4-ethyloctanoic acid and its efficacy under complex environmental conditions require further investigation.

## 4. Materials and Methods

### 4.1. Materials

The five invasive alien plants were all provided by the Hunan Academy of Agricultural Sciences, and the *A*. *frigida* was collected from grassland near Mapisi Temple, Haibei Prefecture, Qinghai Province (Longitude: 100.85331594; Latitude: 37.01392568).

### 4.2. Ethanol Extract of A. frigida

The aboveground parts of *A. frigida* were collected, air-dried, and cut into approximately 1 cm segments. A 50.0 g sample of the dried material was placed in a beaker and extracted with 500 mL of anhydrous ethanol at a solid-to-liquid ratio of 1:10 (*w*/*v*) at 25 °C for 48 h. The extract was filtered under vacuum, and the ethanol filtrate was concentrated by rotary evaporation at 65 °C to remove approximately 95% of the solvent. The concentrated extract was then diluted with sterile distilled water to obtain a final concentration of 50 g·L^−1^, which was stored at −20 °C until further use [32].

### 4.3. Bioassay of A. frigida Extract

Seed germination bioassays were conducted using the filter paper method in Petri dishes. Healthy and uniformly sized seeds of the target weed species were selected and placed in 9 cm Petri dishes lined with two layers of filter paper. And 20–30 seeds were placed in each Petri dish (except for *I. purpurea*, where 10–15 seeds were placed per dish due to their larger seed size). The filter papers were moistened with 5 mL of *A. frigida* ethanol extract at concentrations of 1 g·L^−1^ and 5 g·L^−1^. Distilled water was used as the control (CK). Each treatment was replicated three times. The Petri dishes were placed in an illuminated incubator set at 28 ± 1 °C with a 12 h/12 h light/dark photoperiod. Germination was recorded daily for 7 days, and a seed was considered germinated when the radicle protruded by ≥1 mm. After 7 days, the germination rate of each weed species was determined [20,33].

### 4.4. LC-MS/MS Analysis

Aliquots of 200 µL of extract were transferred to Eppendorf tubes and dried. The dried residues were reconstituted in 200 µL of extraction solution (methanol:acetonitrile:water = 2:2:1, *v*/*v*/*v*) containing deuterated internal standards. The mixture was vortexed for 30 s, sonicated in an ice-water bath for 10 min, and incubated at −40 °C for 1 h. The samples were then centrifuged at 12,000 rpm (13,800× *g*, radius 8.6 cm) for 15 min at 4 °C, and the supernatant was transferred to an autosampler vial for LC-MS/MS analysis.

Chromatographic separation was performed on a Vanquish UHPLC system (Thermo Fisher Scientific, Waltham, MA, USA) equipped with a Phenomenex Kinetex C18 column (2.1 mm × 50 mm, 2.6 µm). The mobile phase consisted of (A) 0.01% acetic acid in water and (B) isopropanol:acetonitrile (1:1, *v*/*v*). The column temperature was maintained at 25 °C, the autosampler temperature at 4 °C, and the injection volume was 2 µL. Mass spectrometry was performed using an Orbitrap Exploris 120 mass spectrometer (Thermo Fisher Scientific) operating in information-dependent acquisition (IDA) mode under the control of Xcalibur software (version 4.4, Thermo Fisher Scientific). The ESI source conditions were as follows: sheath gas flow rate, 50 Arb; auxiliary gas flow rate, 15 Arb; sweep gas, 1 Arb; capillary temperature, 320 °C; vaporizer temperature, 350 °C; spray voltage, 3.8 kV (positive) or −3.4 kV (negative). Full MS scans were acquired at a resolution of 60,000, and MS/MS scans at 15,000 with stepped normalized collision energies (SNCE) of 20, 30, and 40 eV. The raw data were converted to mzXML format using ProteoWizard software (version 3.0.24054) and processed with an in-house R package based on XCMS for feature detection, extraction, alignment, and integration. Metabolite annotation was achieved by matching the acquired MS/MS spectra against an in-house secondary mass spectral database (BiotreeDB V3.0 and BT-Plant V1.1), and the resulting list of compounds present in the ethanol extract of *A. frigida* was compiled accordingly.

### 4.5. Effects of Compounds from A. frigida on Seed Germination of Five Invasive Alien Plants

Based on a qualitative matching score greater than 3.9 from LC-MS/MS spectra, thirteen compounds with relatively high content were selected. Standard samples of these compounds were purchased from Shanghai Macklin Biochemical Technology Co., Ltd. (Shanghai, China, https://www.macklin.cn), and dissolved in dimethyl sulfoxide, water or ethanol, respectively, and then diluted with distilled water to prepare 1 g·L^−1^ stock solution. The stock solution was further diluted with distilled water to concentrations of 0.1 and 0.5 g·L^−1^ for herbicidal activity screening. Seed germination bioassays were conducted using the filter paper method in Petri dishes. Healthy and uniformly sized seeds (20–30 per dish) of the target plants were selected and placed in Petri dishes (9 cm in diameter) lined with two layers of filter paper. 5 mL of each compound solution at the designated concentrations was added to each Petri dish, with an equal volume of distilled water used as the control (CK). Each treatment was replicated three times. The Petri dishes were placed in an illuminated incubator set at 28 ± 1 °C with a 12 h/12 h light/dark photoperiod. After 7 days, the root length and shoot length of each plant were measured [20]. The aim was to identify allelopathic substances from the ethanol extract of *A. frigida* that inhibit the seed germination of invasive alien weeds.

### 4.6. Effects of Selected Allelochemicals on Seedling Growth of Five Invasive Alien Plants

Based on the results of the above experiments, four allelopathic substances with relatively high herbicidal activity, namely, 4-ethyloctanoic acid, cis-jasmone, and p-anisic acid, were selected. Their treatment concentrations were reduced to 10, 30, and 50 mg·L^−1^, and the herbicidal activity of these major allelopathic substances was further evaluated following the same procedure as described above. The inhibition rate was calculated according to the following Equation (1).

(1) Inhibition rate(%) = (CK − T)/CK × 100,

CK: mean root or shoot length of plants grown in the control, T: root length or shoot length of the treatment group.

### 4.7. Effects of 4-Ethyloctanoic Acid on Antioxidant Enzyme Activities of Five Invasive Alien Plants

Following pot spray treatment, the activities of CAT, POD, and SOD were determined using corresponding assay kits (Abbkine Scientific Co., Ltd., Wuhan, China, https://www.abbkine.cn). Antioxidant enzyme activities were calculated according to the following Equations (2)–(4)

(2) POD (U/g) = (A_90s_ − A_30s_) × V1 ÷ (W ÷ V2 × V3) ÷ 0.005 ÷ T,

(3) SOD (U/g) = [(ΔA1 − ΔA2) ÷ ΔA1 × 100% ÷ (1 − ((ΔA1 − ΔA2) ÷ ΔA1 × 100%) × V1] ÷ (W × V3 ÷ V2),

(4) CAT (U/g) = y ÷ T ÷ W = 8.5 × y ÷ 20 ÷ W

A_30s_, A_90s_: Absorbance values at 30 s and 90 s measured at a wavelength of 470 nm; V1: Total volume of the reaction system; V2: Volume of extraction solution added; V3: Volume of sample added; T: Reaction time; W: Sample weight; ΔA1: Absorbance of the blank minus absorbance of the blank control, measured at 450 nm; ΔA2: Absorbance of the sample minus absorbance of the sample control, measured at 450 nm; y: Formaldehyde concentration of the sample calculated from the standard curve.

### 4.8. Statistical Analysis

Three biological replicates were collected from every treatment group, and the data are presented as the mean ± standard error for the three replicates. All data were analyzed using R software (version 4.3.1). Figures were created with Origin 2021 (Origin Lab Corporation, Northampton, MA, USA).

## 5. Conclusions

This study indicates that *A. frigida* exhibits strong allelopathic potential against multiple invasive alien plants. Ethanol extract of *A. frigida* inhibited seed germination and seedling growth of five tested species in a concentration-dependent manner, with complete inhibition observed at 5 g·L^−1^. LC-MS/MS analysis identified over 1700 compounds in the extract, among which 4-ethyloctanoic acid, cis-jasmone, and p-anisic acid were found to exert pronounced allelopathic effects. Notably, 4-ethyloctanoic acid (L1) displayed broad-spectrum inhibitory activity, significantly reducing germination and growth of multiple species, and was further shown to disrupt the redox balance in plant cells by modulating the activities of key antioxidant enzymes (SOD, POD, and CAT). Species-specific antioxidant responses revealed differential strategies in coping with oxidative stress, highlighting the role of 4-ethyloctanoic acid in weed defense systems. These findings indicate that 4-ethyloctanoic acid is a main allelochemical responsible for the herbicidal activity of *A. frigida*, offering potential for the development of natural herbicides. Nevertheless, further studies are warranted to elucidate its precise molecular targets and evaluate its efficacy under field conditions.

## Figures and Tables

**Table 1:** Compounds in the ethanol extract of *A. frigida*.

NO.	Name	MS2 Score	Mz (m/z)	Rt (s)	CAS	Relative Content (%)
L1	4-Ethyloctanoic acid	3.97	171.1393	274.3	16493-80-4	0.40%
L2	Dodecanoic acid	3.96	199.1707	296.4	143-07-7	0.05%
L3	Adipic acid	3.95	145.0508	42.8	124-04-9	0.01%
L4	cis-Jasmone	3.94	165.1275	243.5	488-10-8	0.30%
L5	3,5-Dimethoxycinnamic acid	3.93	191.0704	204.5	16909-11-8	0.01%
L6	3,4-Dihydroxybenzoic acid (Protocatechuic acid)	3.93	153.0195	46.6	99-50-3	0.04%
L7	3-Hydroxyacetophenone	3.93	135.0453	164.5	121-71-1	0.15%
L8	4′-Hydroxyacetophenone	3.93	135.0453	164.5	99-93-4	0.15%
L9	Camphor	3.92	153.1276	239.4	76-22-2	0.53%
L10	N-Acetylvaline	3.92	158.0824	40.1	96-81-1	0.01%
L11	3-(2,5-Dimethoxyphenyl)propionic acid	3.92	209.0822	197.5	10538-49-5	0.05%
L12	p-Anisic acid	3.91	151.0403	196.2	100-09-4	2.19%
L13	4-Hydroxybenzaldehyde	3.9	121.0297	93.8	123-08-0	0.01%

The MS2 score represents the score for secondary matching, with higher values being better; Mz refers to the mass-to-charge ratio of the characteristic ion of a substance; Rt denotes the chromatographic retention time of the substance; relative content indicates the proportion of a particular substance in the total amount; CAS is the Chemical Abstracts Service registry number.

**Table 2:** Effects of compounds on seed germination of five invasive alien plants.

Treatment	Concentration /g·L^−1^	Germination Rate/%				
		*I. purpurea*	*B. alba*	*A. conyzoides*	*A. retroflexus*	*E. prostrata*
	0	96.67 ± 3.33 a	90.00 ± 5.77 a	96.67 ± 3.33 a	72.98 ± 3.56 bcde	55.28 ± 3.74 cdefg
L1	0.1	70.83 ± 11.02 abcdef	0.00 ± 0.00 i	42.93 ± 4.97 def	0.00 ± 0.00 i	0.00 ± 0.00 l
	0.5	0.00 ± 0.00 i	0.00 ± 0.00 i	0.00 ± 0.00 g	0.00 ± 0.00 i	0.00 ± 0.00 l
	1	0.00 ± 0.00 i	0.00 ± 0.00 i	0.00 ± 0.00 g	0.00 ± 0.00 i	0.00 ± 0.00 l
L2	0.1	80.00 ± 5.77 abcd	44.44 ± 12.73 def	98.04 ± 3.39 a	81.01 ± 6.65 abc	66.87 ± 19.91 bcd
	0.5	73.33 ± 6.67 abcde	3.03 ± 5.25 i	88.89 ± 10.18 a	10.67 ± 1.75 i	22.84 ± 5.07 jk
	1	53.33 ± 12.02 ef	0.00 ± 0.00 i	54.45 ± 10.72 cde	0.00 ± 0.00 i	0.00 ± 0.00 l
L3	0.1	90.47 ± 4.76 abc	63.64 ± 13.64 bcd	73.33 ± 12.02 abc	68.8.0 ± 11.68 cdef	35.65 ± 11.68 ij
	0.5	85.71 ± 8.25 abcd	16.67 ± 12.02 ghi	74.24 ± 14.45 abc	40.71 ± 4.67 gh	0.00 ± 0.00 l
	1	14.29 ± 8.25 hi	0.00 ± 0.00 i	0.00 ± 0.00 g	0.00 ± 0.00 i	0.00 ± 0.00 l
L4	0.1	62.5 ± 14.43 def	0.00 ± 0.00 i	73.33 ± 12.02 abc	0.00 ± 0.00 i	0.00 ± 0.00 l
	0.5	0.00 ± 0.00 i	0.00 ± 0.00 i	0.00 ± 0.00 g	0.00 ± 0.00 i	0.00 ± 0.00 l
	1	0.00 ± 0.00 i	0.00 ± 0.00 i	0.00 ± 0.00 g	0.00 ± 0.00 i	0.00 ± 0.00 l
L5	0.1	29.17 ± 4.17 gh	10.00 ± 5.77 hi	63.33 ± 17.64 bcd	29.7 ± 2.46 h	18.54 ± 6.42 k
	0.5	0.00 ± 0.00 i	0.00 ± 0.00 i	0.00 ± 0.00 g	0.00 ± 0.00 i	0.00 ± 0.00 l
	1	0.00 ± 0.00 i	0.00 ± 0.00 i	0.00 ± 0.00 g	0.00 ± 0.00 i	0.00 ± 0.00 l
L6	0.1	90 ± 5.77 abc	85.1 ± 3.28 a	87.04 ± 6.68 ab	87.76 ± 3.86 a	80.51 ± 16.06 ab
	0.5	83.33 ± 3.33 abcd	79.55 ± 5.72 ab	87.78 ± 4.21 ab	87.93 ± 2.98 a	85.07 ± 3.54 a
	1	73.33 ± 17.64 abcde	0.00 ± 0.00 i	0.00 ± 0.00 g	79.54 ± 6.24 abc	3.52 ± 3.06 l
L7	0.1	73.33 ± 3.33 abcde	42.68 ± 12.44 ef	93.18 ± 3.85 a	77.59 ± 0.29 abc	69.28 ± 3.64 bc
	0.5	93.33 ± 3.33 ab	0.00 ± 0.00 i	93.33 ± 3.85 a	4.56 ± 2.03 i	0.00 ± 0.00 l
	1	76.67 ± 8.82 abcde	0.00 ± 0.00 i	22.86 ± 2.86 fg	0.00 ± 0.00 i	0.00 ± 0.00 l
L8	0.1	86.67 ± 3.33 abcd	26.77 ± 5.69 fgh	84.29 ± 5.76 ab	76.59 ± 1.3 abcd	53.49 ± 2.06 defgh
	0.5	70.00 ± 15.28 bcde	0.00 ± 0.00 i	87.36 ± 9.50 ab	6.15 ± 1.66 i	0.00 ± 0.00 l
	1	76.67 ± 8.82 abcde	0.00 ± 0.00 i	48.89 ± 22.55 de	0.00 ± 0.00 i	0.00 ± 0.00 l
L9	0.1	90.47 ± 4.76 abc	73.33 ± 3.33 abc	89.43 ± 1.87 a	59.36 ± 3.10 f	44.84 ± 7.1 fghi
	0.5	42.86 ± 14.28 fg	20.00 ± 10 ghi	90.00 ± 5.77 a	47.83 ± 14.56 g	19.6 ± 6.25 k
	1	23.81 ± 4.76 ghi	30.00 ± 10 fgh	40.00 ± 5.77 ef	7.90 ± 4.44 i	0.00 ± 0.00 l
L10	0.1	90.48 ± 9.52 abc	79.80 ± 10.25 ab	80.73 ± 5.66 ab	85.23 ± 4.11 ab	62.04 ± 10.11 cde
	0.5	85.71 ± 8.25 abcd	56.67 ± 23.33 cde	85.86 ± 9.95 ab	69.91 ± 6.75 cdef	39.66 ± 6.74 hi
	1	85.71 ± 0.00 abcd	56.67 ± 6.67 cde	76.16 ± 4.78 abc	80.00 ± 2.89 abc	45.53 ± 6.25 fghi
L11	0.1	90.00 ± 0.00 abc	25.76 ± 4.87 fgh	87.45 ± 3.37 ab	63.79 ± 5.17 def	58.66 ± 7.97 cdef
	0.5	66.67 ± 8.82 cde	0.00 ± 0.00 i	13.33 ± 13.33 g	2.53 ± 1.59 i	0.00 ± 0.00 l
	1	26.67 ± 12.02 gh	0.00 ± 0.00 i	0.00 ± 0.00 g	0.00 ± 0.00 i	0.00 ± 0.00 l
L12	0.1	91.67 ± 4.17 abc	0.00 ± 0.00 i	90.00 ± 5.77 a	68.61 ± 0.32 cdef	33.74 ± 8.97 ij
	0.5	16.67 ± 11.02 hi	0.00 ± 0.00 i	0.00 ± 0.00 g	0.00 ± 0.00 i	0.00 ± 0.00 l
	1	0.00 ± 0.00 i	0.00 ± 0.00 i	0.00 ± 0.00 g	0.00 ± 0.00 i	0.00 ± 0.00 l
L13	0.1	95.83 ± 4.17 ab	86.67 ± 6.67 a	96.67 ± 3.33 a	80.27 ± 7.24 abc	42.5 ± 6.09 ghi
	0.5	87.5 ± 0.00 abcd	36.67 ± 6.67 efg	50.00 ± 0.00 de	60.01 ± 3.55 ef	51.39 ± 1.95 efgh
	1	71.76 ± 4.70 abcde	0.00 ± 0.00 i	0.00 ± 0.00 g	0.00 ± 0.00 i	0.00 ± 0.00 l

Data are expressed as the mean ± SE, and different letters indicate significant differences between different treatments (*p* < 0.05).

## Data Availability

The data presented in this study are available on request from the corresponding author. The data are not publicly available due to privacy.
